# Acupuncture Synergized With Bortezomib Improves Survival of Multiple Myeloma Mice *via* Decreasing Metabolic Ornithine

**DOI:** 10.3389/fonc.2021.779562

**Published:** 2021-11-02

**Authors:** Mengying Ke, Jinjun Qian, Feng Hao, Xinying Li, Hongjie Wu, Xian Luo, Bin Xu, Chunyan Gu, Ye Yang

**Affiliations:** ^1^ Large Data Center, Nanjing Hospital of Chinese Medicine Affiliated to Nanjing University of Chinese Medicine, Nanjing, China; ^2^ School of Medicine & Holistic Integrative Medicine, Nanjing University of Chinese Medicine, Nanjing, China; ^3^ Acupuncture and Tuina College, Nanjing University of Chinese Medicine, Nanjing, China; ^4^ Key Laboratory of Acupuncture and Medicine Research of Ministry of Education, Nanjing University of Chinese Medicine, Nanjing, China

**Keywords:** ornithine, metabolomics, multiple myeloma, acupuncture, ODC1

## Abstract

Multiple myeloma (MM) is a hematological malignancy worldwide in urgent need for novel therapeutic strategies. Since Velcade (bortezomib) was approved for the treatment of relapsed/refractory MM in 2003, we have seen considerable improvement in extending MM patient survival. However, most patients are fraught with high recurrence rate and incurability. Acupuncture is known for alleviating patient symptoms and improving the quality of life, but it is not well investigated in MM, especially in combination with bortezomib. In this study, we employed LC-MS and UHPLC-MS together with bioinformatics methods to test serum samples from 5TMM3VT MM murine model mice with four different treatments [control (C) group, bortezomib (V) treatment group, acupuncture (A) group, and combined (VA) group]. MM mice in group VA had longer survival time than mice in group A or group V. Joint pathway analysis indicated the underlying arginine and proline metabolism pathway among the 32 significantly decreased metabolites in group VA. CCK-8 assay and *in vivo* experiments validated that ornithine, the metabolite of arginine, promoted MM cell proliferation. In addition, gene expression omnibus (GEO) database analysis suggested that MM patients with higher ornithine decarboxylase 1 (ODC1) expression were evidently associated with poor overall survival. In summary, this study demonstrates the synergistic effects of acupuncture and bortezomib on extending the survival of MM model mice and provides potential therapeutic targets in the treatment of MM.

## Introduction

Multiple myeloma (MM) is a hematological malignancy with clonal proliferation of abnormal plasma cells in the bone marrow (1, 2). According to the statement of international myeloma working group (IMWG), there were nearly 159,985 new MM patients diagnosed annually worldwide. About 1% of patients with monoclonal gammopathy of undetermined significance (MGUS) progressed to MM every year (3, 4). In 2003, Velcade (bortezomib for injection) was approved by the US Food and Drug Administration for the treatment of relapsed/refractory MM as the first proteasome inhibitor (5). The latest clinical MM therapy is a new immunomodulatory therapy using chimeric antigen receptor T cells, bispecific T cell conjugates, and immune checkpoint inhibitors (6). Although the therapeutic armamentarium for MM has continued to evolve (7–14), MM still possesses the characteristics of high relapse and incurability. It is necessary to explore more effective therapies to improve MM prognosis significantly.

In the national comprehensive cancer network (NCCN) (15), adult cancer pain clinical guidelines recommend acupuncture as a comprehensive treatment option in combination with pharmacologic interventions. Nowadays, acupuncture, especially serving as a non-drug alternative to control symptoms, has become a popular adjuvant therapy in cancer treatment (16, 17). Many clinical cases reported that the combination of acupuncture and medicine not only delayed the disease progress (18), reduced the dosage (19), and minimized the side effects of the drug (20), but also relieved the pain caused by the diseases (21, 22). Therefore, the combined application of acupuncture and bortezomib in MM might have broad prospect in alleviating patient symptoms and improving the quality of life.

Since Warburg and Cori demonstrated that cancer cells increased glucose uptake and the fermentation of glucose into lactic acid to promote cellular growth, survival, and proliferation (termed “Warburg effect”) in the 1920s (23), metabolic reprogramming was deemed as one of the main hallmarks and adaptive phenotypes exploited by tumor cells during all the tumor growth and metastatic progression (24), such as abnormal glucose metabolism in colorectal cancer (25), acute myeloid leukemia accompanied by abnormal glycolysis (26), lipid metabolism disorders in hepatocellular carcinoma (27), and cytochrome P450-derived arachidonic acid metabolism in pheochromocytoma (28). Modern metabolomics techniques utilize nuclear magnetic resonance or chromatography mass spectrometry to detect differential metabolites in serum and analysis of metabolic profiles.

To explore the effects of acupuncture combined with bortezomib (VA) in MM and the underlying potential mechanism, we performed chromatography–mass spectrometry (LC-MS) and UHPLC-MS together with bioinformatics, joint pathway analysis, and gene expression profiling (GEP) analysis to determine the metabolomics in MM serum samples.

## Methods

### Chemicals and Reagents

The 5TMM3VT murine myeloma cells were donated by Professor Wen Zhou from the State Key Laboratory of Experimental Hematology, Department of Hematology, Xiangya Hospital, Central South University. Velcade (bortezomib for injection) was purchased from Hansoh pharma (H20173307, Jiangsu, China). Acetonitrile and methanol were purchased from Merck, Millipore Ltd (1.00030.4008, 1.06007.4008, Carrigtwohill, Ireland). Formic acid, ethyl acetate, trifluoroacetic acid, ammonium acetate, and L-arginine hydrochloride were purchased from Macklin Biochemical Co., Ltd (F809712, E809174, T818778, A801000, L800291, Shanghai, China). The 2-chloro-L-phenylalanine and dansyl chloride were purchased from Yuanye Bio-Technology Co., Ltd (B25643, S19248, Shanghai, China). The 1,4-Butane-1,1,2,2,3,3,4,4-d8-diamine was purchased from Toronto Research Chemicals (D416027, Canada). Chloroform, sodium carbonate, sodium bicarbonate, and acetone were purchased from Lingfeng Chemical Reagent Co. (Shanghai, China). Ornithine analytical standard was purchased from Solarbio (SO8470, Beijing, China). RPMI-1640 medium without arginine was purchased from Sigma-Aldrich (R1780, USA). Certified fetal bovine serum, RPMI 1640 medium, and dialyzed fetal bovine serum were purchased from Biological Industries (05-065-1A, 04-002-1A, 04-011-1b, Kibbutz Beit Haemek, Israel). Cell counting kit (CCK-8) was purchased from Yeasen Biotechnology Co., Ltd. (40203ES76, Shanghai, China).

### Animal Protocols

All animal procedures were conducted in accordance with government-published recommendations for the Care and Use of Laboratory Animals and approved by the Institutional Ethics Review Boards of Nanjing University of Chinese Medicine. The experimental mice (C57BL/KaLwRij, 6–8 weeks, 18–20 g) were purchased from the institute of model animals of Nanjing University. The experimental mice were housed in the SPF laboratory animal center of Nanjing University of Chinese Medicine with 15–25°C ambient temperature and free access to food and water. After 1 week of adaptive feeding, animal experiments were started. 5TMM3VT murine myeloma cells (1×10^6^) were injected *via* the tail vein into 6-week-old C57BL/KaLwRij mice. The mice were divided into four groups as follows: model control (C, only modeling, n=10) group, bortezomib (V, n=10) treatment group, acupuncture (A, n=9) treatment group, and VA (n=8) treatment group. One day later, mice in three treatment groups were treated by different methods, containing intraperitoneal injection of 1.2 mg/ml V twice a week or/and electroacupuncture (Model SDZ-II, Suzhou Medical Appliance Factory, Suzhou, China) stimulation of Hegu (29) and Zusanli (30, 31) points (2/100 Hz, 2 mA) three times a week until all the mice were dead.

### Serum Sample Collection

Blood was taken from tail vein on Tuesday and Wednesday during the experimental period. Blood samples of week 4–6 were mixed in the clean Eppendorf tubes, stored on ice for 2 h, and centrifuged (5,000 rpm, 10 min) at 4°C. The blood was collected for separation of serum. Subsequently, the supernatants were transferred to clean Eppendorf tubes and stored at −80°C.

### Sample Preparation for LC-MS Analysis

All serum samples were thawed on the ice. An aliquot of 45 μl serum sample was precipitated by adding 135 μl acetonitrile containing internal standards (IS) [2-Chloro-L-Phenylalanine (plasma sample-acetonitrile: IS=2,000:1)], vortex for 30 s, sonicating for 10 min at 4°C, and then stayed for 3 h on ice. Precipitated protein was removed by centrifugation (13,000 rpm, 10 min) at 4°C. Subsequently, 153 μl supernatant was transferred to glass inserts of LC-MS vials and stored at −80°C for LC-MS analysis.

### LC-MS Analysis

The LC-300AD LC system (Shimadzu, Japan) coupled to a Triple TOF™5600 mass spectrometer (AB SCIEX, USA) and operated in full scan mode was used for untargeted analysis of serum samples. Each sample was run in duplicate in electron spray ionization^+/−^ (ESI^+/−^) modes. An aliquot of 3 μl extracted plasma sample was injected onto an ACQUITY UPLC HSS T3 C18 (2.1 × 100 mm, 1.8 μm) column (Waters, USA) operating at 40°C. The auto-sampler was conditioned at 4°C. Untargeted metabolomics were detected as described in a previous study (6).

Raw data files from LC-MS were converted by Analyst^®^TF 1.7 software and imported into Markview software to match the peaks and the metabolites identified by mass spectral database of Dalian Institute of Chemical Physics, Chinese Academy of Sciences. Mass-to-charge ratio difference less than 0.001 was regarded as the same substance. Then the dataset of normalized peak height intensity, retention time (RT), metabolites names, and sample numbers were analyzed by SIMCA 14.1 software. SIMCA 14.1 conducted a multivariate statistical analysis of the principal component analysis (PCA) and orthogonal partial least-squares discrimination analysis (OPLS-DA) and permutations. The metabolites with P value < 0.05 and variable importance in the projection (VIP) of >1.0 were considered as statistically significant metabolites. The metabolic joint pathway analysis was carried out on the website visualization tools of MetaboAnalyst 5.0 (32).

### Sample Preparation for UHPLC-MS Analysis

Five serum samples were randomly selected from group C and group VA, respectively, and prepared for UHPLC-MS analysis. All serum samples were thawed on the ice. An aliquot of 50 μl serum sample was precipitated by adding 5 μl 1,4-Butane-1,1,2,2,3,3,4,4-d8-diamine and 167 μl methanol, vortex for 1 min, then adding 334 μl chloroform and vortex again for 1 min. Supernatants were collected by centrifugation (15,000 rpm, 10 min) at 4°C. Then 100 μl sodium bicarbonate-sodium bicarbonate buffer (pH=9) and 50 μl dansyl chloride solution (dissolved in acetone) were added to the supernatant (33), and stayed for 1 h at room temperature in dark area. Subsequently, the organic phase was extracted with acetic ether twice. Notably, trifluoroacetic acid was added before the second extraction. Finally, the organic phase was transferred to fresh tube and dried in solvent evaporator (Genevac, UK) at 45°C for 2 h. The residue was reconstituted in 100 μl of a mixture of 0.2 mol/L ammonium acetate/acetonitrile (3:7, vol/vol) for UHPLC-MS analysis.

### UHPLC-MS Analysis

Waters iClass UHPLC system (Waters, USA) coupled with a Triple Quad™ 6500+ (AB SCIEX, USA) was applied for targeted metabolomics analysis. An aliquot of 1 µl sample solution was injected onto Ultimate XB-AQ chromatographic column (100 mm × 2.1 mm, 3 μm) maintained at 40°C. The auto-sampler was conditioned at 4°C. For carrying out analysis, the mobile phase was composed of A (0.1% formic acid in water) and B (acetonitrile acidified by 0.1% formic acid) with different concentration gradient. The flow rate was 0.4 ml/min. Mass spectrometric (MS) parameters were applied as follows: ionization temperature 450°C, ion-source gas 1 pressure 55 psi, ion-source gas 2 pressure 55 psi, curtain gas pressure 40 psi, and ion-source voltage 5,500 V.

### Cell Proliferation Assay

Cell growth was evaluated by using CCK8 assay according to the method described in the literature (34). Cells were cultured for 24 h with dialyzed fetal bovine serum and RPMI-1640 medium without arginine, then seeded at a density of 1,500 cells/well in 96-well plates. MM cells were cultured with different concentrations of arginine for 24 and 72 h. And 10 µl CCK8 was added to each well for 3 h before detection. The absorbance was measured at A450 nm with a microplate plate reader (Thermo Fisher Scientific, Inc., USA).

### Statistics Analysis

Survival analyses were made by using the Kaplan Meier method. Statistical analyses were performed by using GraphPad Prism 8 software. The statistical results were conducted with Log-rank (Mantel-Cox), and value of P< 0.05 was regarded as a significant difference (*P<0.05, **P<0.01, ***P<0.001).

## Results

### Efficacy Evaluation of VA Treatment in 5TMM3VT Myeloma Mice

**Figure 1:**
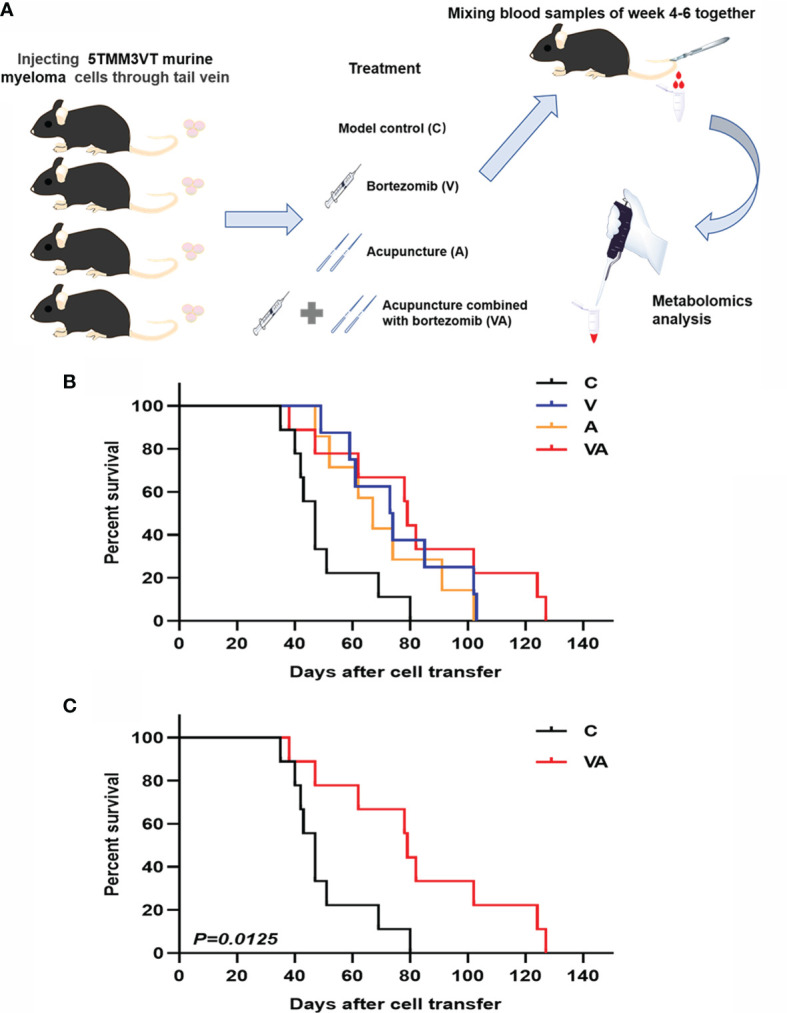
Efficacy evaluation of VA treatment in 5TMM3VT myeloma mice. **(A)** Animal model and blood collection. **(B)** Survival curve of group C, V, A, and VA. **(C)** Survival curve of group C *vs* VA.

The MM mouse model was established by tail vein injection of 5TMM3VT murine myeloma cells (**Figure 1A**) and subjected to four different groups: only modeling (group C), bortezomib treatment (group V), acupuncture treatment (group A), and combination therapy (group VA). As **Figure 1B** shows, the survival rates of myeloma mice in group V (median time of 73.5 days) and A (median time of 67 days) were evidently improved compared with the group C (median survival time of 47 days). Intriguingly, the median survival time of the myeloma mice treated with VA significantly prolonged to 79 days, and in the sixth week after modeling, the survival curves began to show significant differences between the group VA and the group C (**Figure 1C**).

### Serum Metabolic Profiling Reveals Significant Differences Among MM Mice in Different Treatment Groups

**Figure 2:**
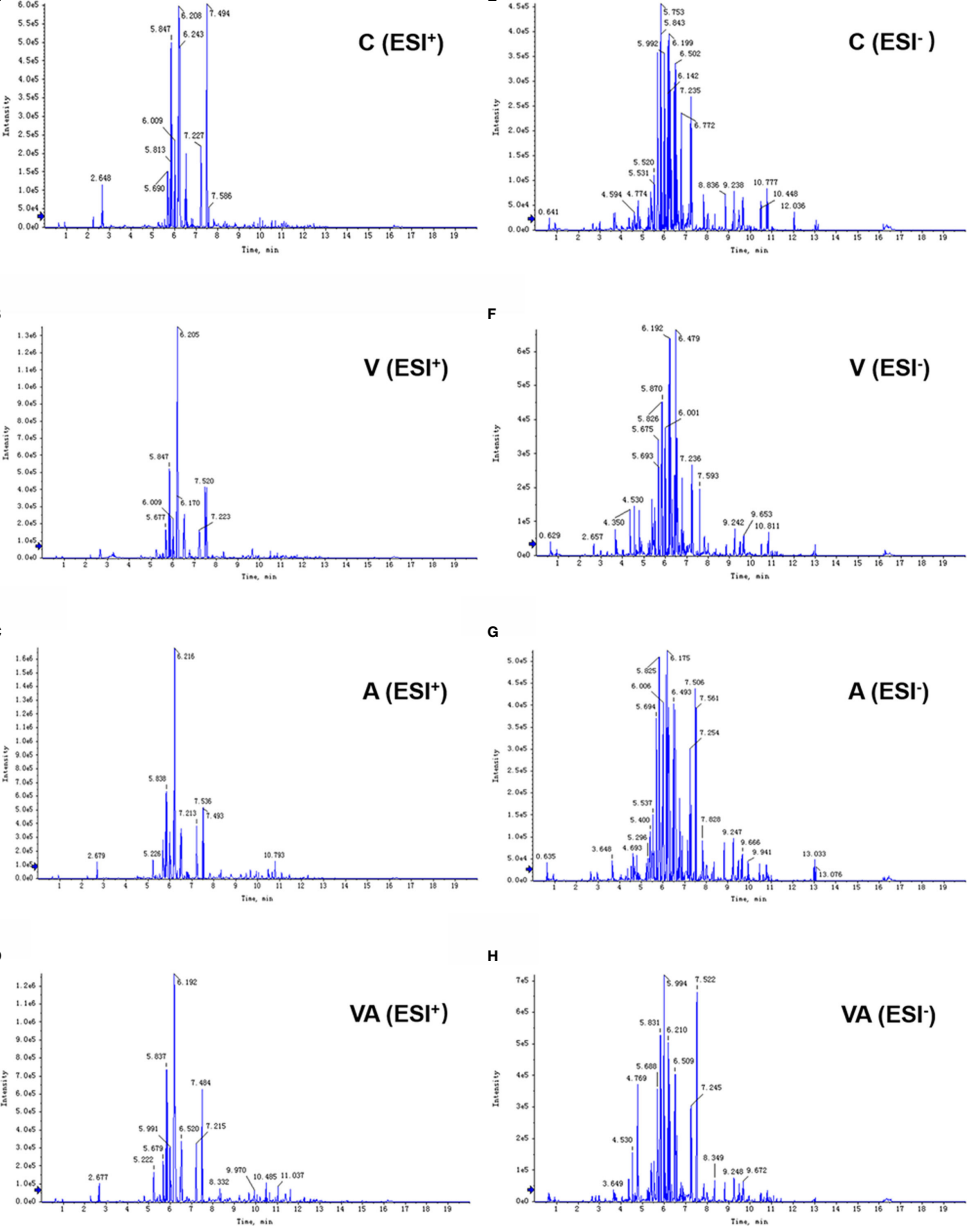
Typical chromatograms of TIC in serum samples. TIC of group C **(A)**, V **(B)**, A **(C)**, and VA **(D)** in ESI^+^ mode. TIC of group C **(E)**, V **(F)**, A **(G)**, and VA **(H)** in ESI^−^ mode.

Serum was collected from the myeloma mice in each group, which was used to examine the characteristics of metabolites by LC-MS. The results showed that the peak patterns of total ion current (TIC) obtained in ESI^+^ (**Figures 2A–D**) and ESI^−^ (**Figures 2E–H**) modes were distinctly different. The serum metabolites of the MM mice in each group were well separated under the same detection mode. Within 5~13 min of the injection, there were significant differences between group C and groups A, V, or VA.

**Figure 3:**
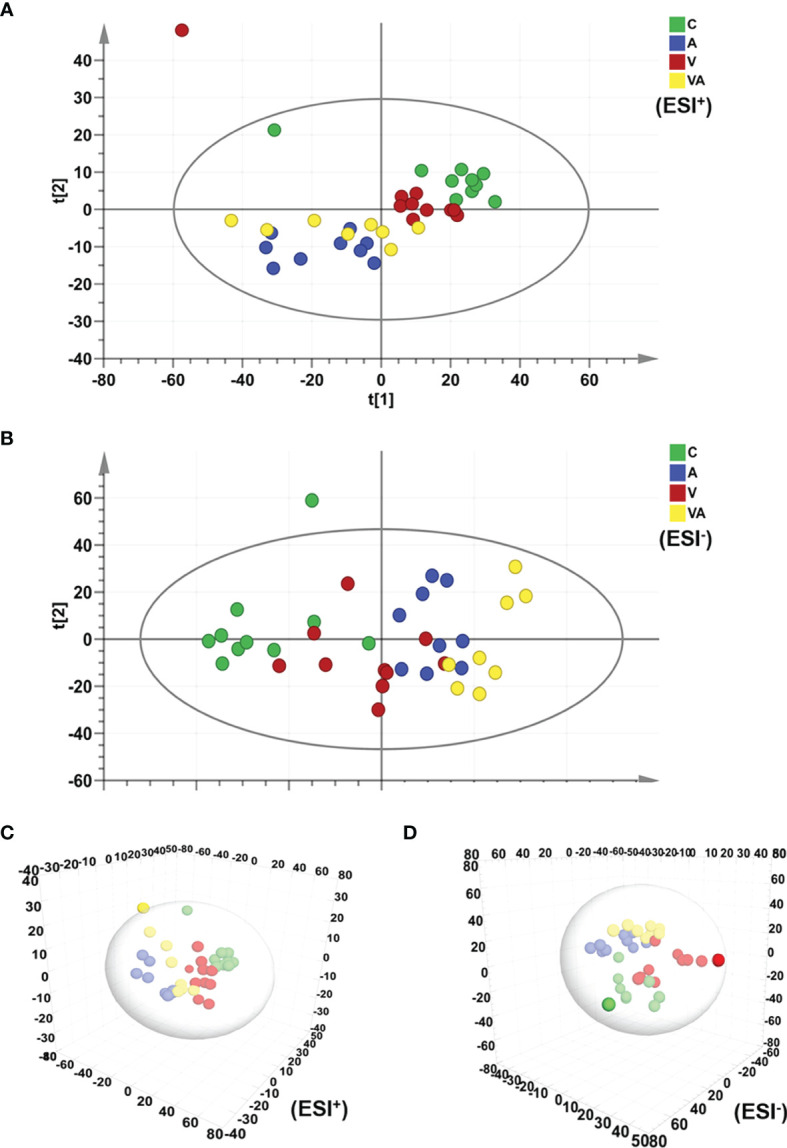
PCA score plot based on the data of ESI^+/−^ modes. **(A)** PCA score plot of all groups in ESI^+^ mode. **(B)** PCA score plot of all groups in ESI^−^ mode. **(C)** 3D scatter plot of all groups in ESI^+^ mode. **(D)** 3D scatter plot of all groups in ESI^-^ mode.

The principal component analysis (PCA) was used to reflect the degree of dispersion among the four groups. Differences and changes in metabolic profiles of MM mouse serum from each group were evaluated by PCA in ESI^+^ (**Figure 3A**) and ESI^−^ (**Figure 3B**) modes. The results displayed a significant separation of serum samples from mice in the four groups with good clustering of samples within groups (**Figures 3A, B**), as well as the three-dimensional (3D) scatter plot (**Figures 3C, D**).

**Figure 4:**
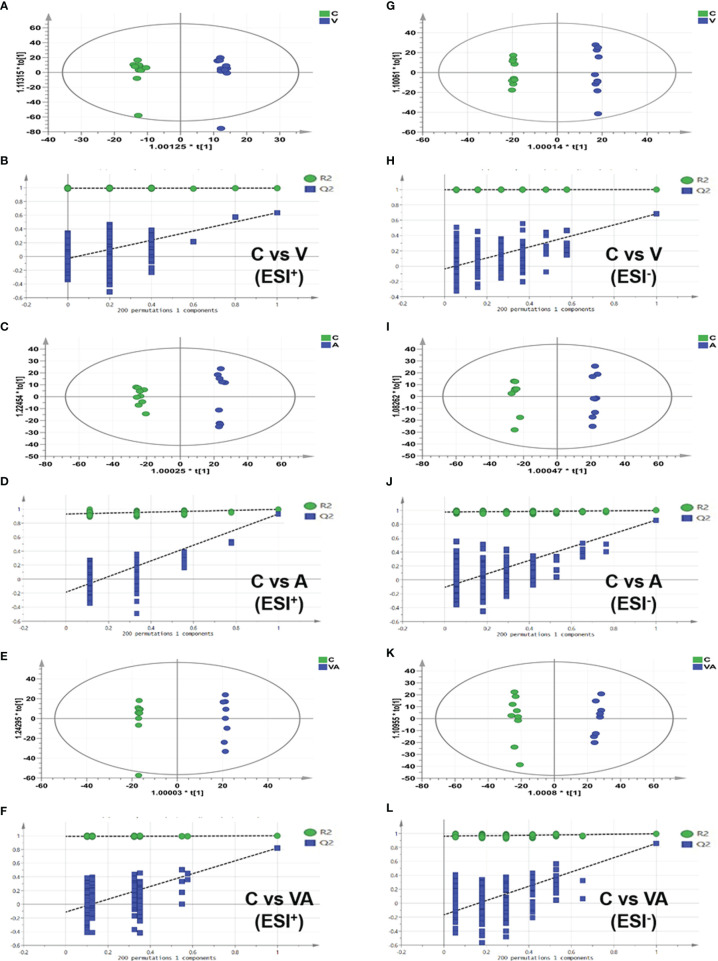
OPLS-DA score plot based on the data of ESI^+/−^ modes and validations of OPLS-DA models by 200 permutation tests. In ESI^+^ mode: **(A, B)** group C *vs* group V, **(C, D)** group C *vs* group A, **(E, F)** group C *vs* group VA. In ESI^−^ mode: **(G, H)** group C *vs* group V, **(I, J)** group C *vs* group A, **(K, L)** group C *vs* group VA.

The orthogonal partial least-squares discrimination analysis (OPLS-DA) model of serum metabolomics from myeloma mice showed the significant differences in group V, A, or VA compared with group C in both ESI^+^ (**Figures 4A, C, E**) and ESI^−^ (**Figures 4G, I, K**) modes. In addition, all the permutation test results indicated that the fitted model was reliable (**Figures 4B, D, F, H, J, L**). The differential metabolites that satisfied the criterion (VIP >1.0 and P value <0.05) were considered as significantly different substances. There were 97 different substances in the serum of group V compared with group C, including 64 upregulated and 33 downregulated substances (**Figures 4A, B, G, H**). There were 151 different serum substances between group A and group C, with 113 upregulated and 28 downregulated substances (**Figures 4C, D, I, J**). Importantly, we found 174 different substances in the serum of group VA in comparison with group C, including 102 upregulated and 72 downregulated substances (**Figures 4E, F, K, L**).

### Ornithine Acts as a Therapeutic Target of VA Treatment in MM Mice

**Figure 5:**
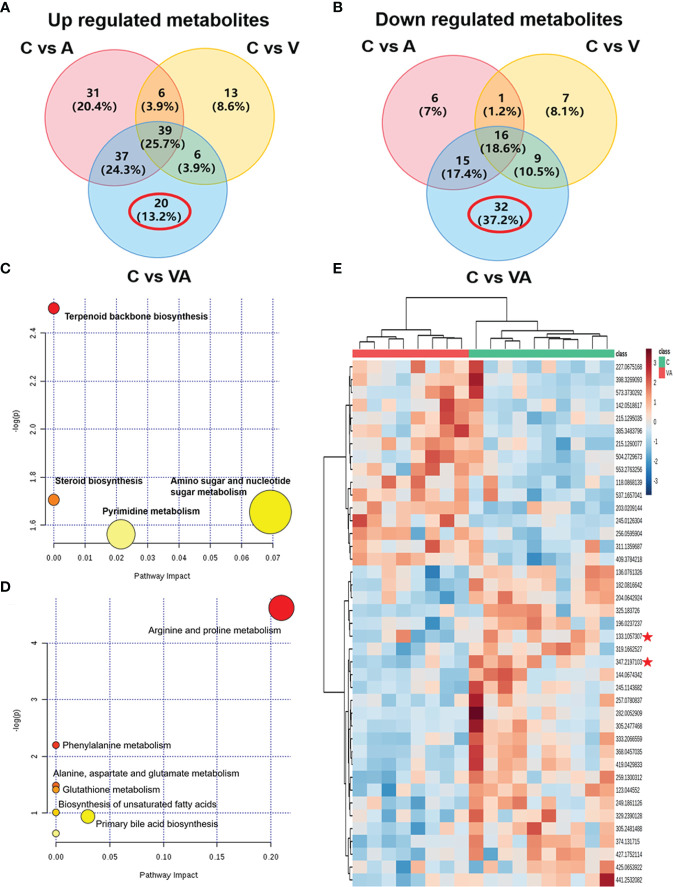
Ornithine is a therapeutic target of VA treatment in MM mice. **(A)** Venn diagram displaying the 20 upregulated distinct metabolites in the serum of Group VA. **(B)** Venn diagram displaying the 32 downregulated distinct metabolites in the serum of Group VA. **(C)** Summary of joint pathway analysis in group VA with MetaboAnalyst 5.0. **(D)** Summary of joint pathway analysis in group VA with MetaboAnalyst 5.0. **(E)** Heatmap showing arginine and ornithine were downregulated metabolites in group VA.

To narrow down the potential therapeutic targets, all the significantly different substances from each comparison groups were collected to plot Venn diagrams. Excluding the intersection, there were 20 upregulated (**Figure 5A** and **Table 1**) and 32 downregulated (**Figure 5B** and **Table 2**) distinct metabolites in the serum of group VA. Subsequently, MetaboAnalyst 5.0 was used to analyze the joint pathways of differential metabolites in ESI^+^ (**Figure 5C**) and ESI^−^ (**Figure 5D**) modes, respectively. In **Figure 5D**, the main pathway of arginine and proline metabolism was involved in the serum of group VA with impact 0.20964 (−log(P)=4.6259). Ornithine and arginine were major metabolites in arginine and proline metabolism pathway.

**Table 1:** Partially* distinct upregulated substances in group VA.

Name	m/z	*P*	FC	VIP
2’-Deoxyuridine	227.0675	0.0091	1.8167	1.3109
4-Cholesten-3-One	385.3484	0.0093	1.8663	1.2221
Calcifediol	398.3269	0.0057	1.689	1.295
D-Desthiobiotin	215.126	0.0013	1.2113	1.3391
Dimethylallyl pyrophosphate	245.0126	0.0017	2.7971	1.4729
D-Norvaline	118.0868	0.0128	1.6716	1.0571
Hypoxanthine-9-β-D-arabinofuranoside	537.1657	0.0104	1.6909	1.1158
L-Altrose	203.0234	<0.0001	2.262	1.5535
Lasalocid	573.373	0.0474	1.9948	1.2608
Leucine enkephalin amide	553.2763	0.0159	1.821	1.3428
N-Acetyl-D-glucosamine	256.0596	0.0118	1.1686	1.2742
N-Methyl-L-glutamic acid	142.0519	0.0179	2.0681	1.1724
Nα-Acetyl-L-arginine	215.1295	0.018	1.3949	1.3476
Taurolithocholic acid	504.273	0.0242	1.6907	1.355
α-Amyrin	409.3784	0.0006	1.6457	1.3498

*This table didn’t list five exogenous compounds, namely, dihydrocapsaicin, benzoic acid, Apramycin, sulfa quinazoline (sulfaquinaoxaline), equol.

**Table 2:** Partially* distinct downregulated substances in group VA.

Name	m/z	*P*	FC	VIP
13-Cis-Acitretin	325.1837	0.0033	0.7025	1.4494
2’-Deoxyguanosine-5’-monophosphate	368.0447	0.0017	0.3811	1.5304
2-Phenylacetamide	136.0766	0.0145	0.7426	1.0975
4-Guanidinobutyric acid	144.0681	0.0133	0.3947	1.222
4-Hydroxybenzaldehyde	123.0449	0.0056	0.5706	1.1689
5-Methyluridine	257.0788	0.0245	0.7067	1.1576
7-Ketodeoxycholic acid	441.2529	0.0499	0.6459	1.206
7z,10z,13z-Hexadecatrienoic Acid	249.1856	0.0152	0.7752	1.2389
All-cis-4,7,10,13,16-docosapentaenoic acid	329.2332	0.0403	0.8578	1.0992
Arginine	347.2198	0.0056	0.897	1.3782
Boc-β-cyano-L-alanine	427.1765	0.029	0.7437	1.1536
Cis-8,11,14-Eicosatrienoic acid	305.2477	0.0188	0.8018	1.2626
D-(+)-Octopine	245.1151	0.0209	0.5288	1.1965
Dl-Tyrosine	182.0812	0.01	0.635	1.1161
Glycochenodeoxycholic acid	484.2898	0.0037	0.2768	1.4485
Haloperidol	374.1322	0.0049	0.6947	1.419
Indole-3-pyruvic acid	204.0638	0.0064	0.5987	1.1311
L-Cysteine-glutathione gisulfide	425.0654	0.0291	0.7847	1.087
Mesterolone	305.248	0.013	0.4361	1.0414
Mucic acid	419.0485	0.0018	0.3993	1.5564
N-Acetylaspartate	196.0235	0.0252	0.7582	1.2739
O-Phospho-L-Tyrosine	282.0055	0.0094	0.5057	1.3553
Ornithine	133.1055	0.0333	0.6108	1.0133
Phe-Phe	311.1348	0.0135	0.8143	1.3764
Prostaglandin B2	333.2069	0.006	0.6439	1.385
β-Zearalenol	319.1662	0.0075	0.6886	1.3195
γ-Glu-Leu	259.1303	0.0166	0.5956	1.2689

*This table didn’t list five exogenous compounds, namely, diazepam, diltiazem diacetate, diflunisal, oxfendazole, diflunisal.

Cluster analysis and heatmap showed that both ornithine (median of m/z=133.1057307) and arginine (median of m/z=347.2197103) were significantly decreased in the serum of group VA compared with group C (**Figure 5E**). To a large degree, these data illustrated that VA treatment inhibited arginine and proline metabolism pathway, thus causing arginine and ornithine reduction. Additionally, ornithine was also involved in the regulation of the glutathione metabolic pathway (−log(P)=41.4122) in **Figure 5D**. These results suggested that ornithine might be a therapeutic target of VA treatment in MM.

### VA Treatment Decreases Ornithine Concentration in the Serum of MM Mice

**Figure 6:**
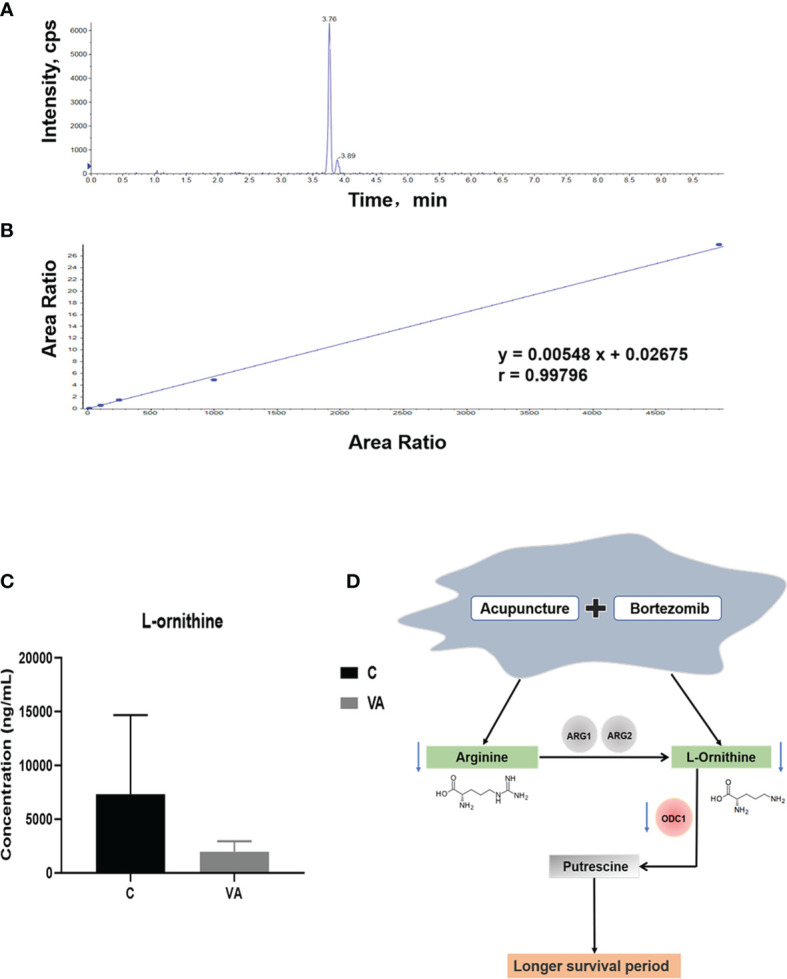
Ornithine concentration is decreased in serum samples of group VA. **(A)** Characteristic chromatographic peak of ornithine standard. **(B)** Standard curve of ornithine in targeted metabolomics. **(C)** Ornithine concentration in serum samples of group C and VA. **(D)** The possible mechanism involved in VA treatment.

To further prove the above data, we conducted targeted metabolomics to detect ornithine concentration in the serum of MM mice. The chromatogram revealed a characteristic peak of ornithine standard at 3.76 min after injection (**Figure 6A**). In **Figure 6B**, according to the linear standard curve (r=0.99796), ornithine content in the serum samples of group VA (Average concentration of 7,333.33 ng/ml) was decreased by 73.36% compared with the group C (Average concentration of 1,953.33 ng/ml) (**Figure 6C**); however, it didn’t reach statistical difference due to the relatively small sample size in each group and large individual variation. In agreement with previous results of untargeted metabolomics, these data confirmed that VA treatment decreased the level of serum ornithine.

### Arginine and Its Metabolite Promote MM Cell Proliferation

**Figure 7:**
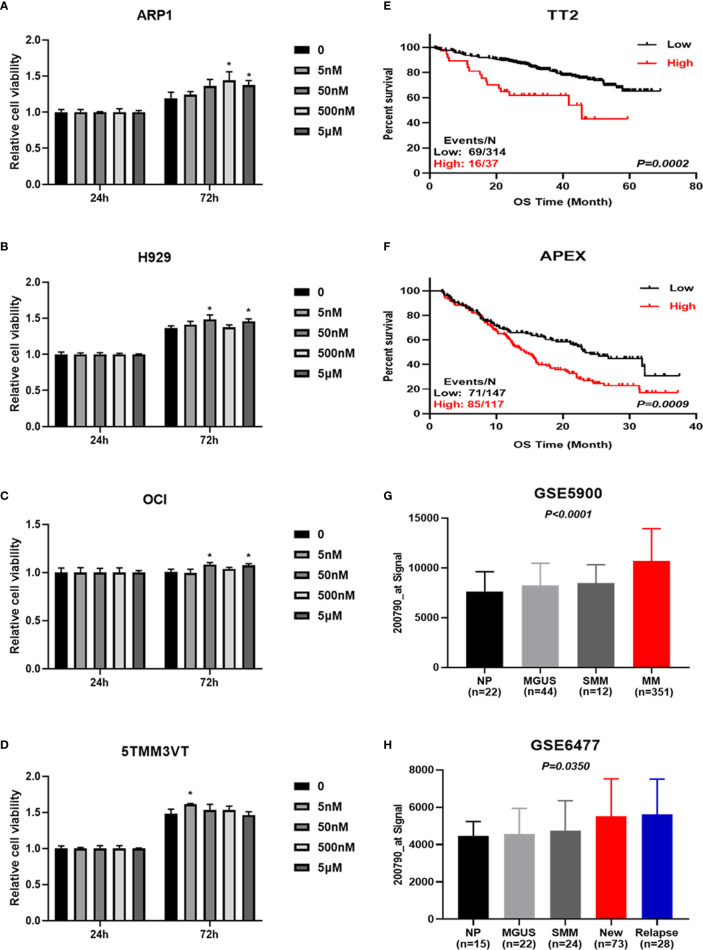
Increased ODC1 expression is associated with poor prognosis in MM. **(A–D)** Arginine and its metabolite promoted ARP1, H929, OCI, and 5TMM3VT cell proliferation. *P < 0.05. **(E, F)** High ODC1 expression in MM patients was correlated with poor OS in TT2 cohort, and APEX phase III clinical trial by log-rank test. **(G, H)** The mRNA level of ODC1 from NP, MGUS, SMM, and MM was significantly increased in MM samples by ordinary one-way ANOVA test.

Arginine is a semi-essential amino acid that can be metabolized into ornithine, which is a non-essential amino acid (**Figure 6D**). We further assessed the effect of supplying extra arginine on MM cell proliferation by using CCK8 assay. As shown in **Figures 7A–D**, the viability of human ARP1, H929, OCI and mouse 5TMM3VT cells was significantly increased upon serial concentration of arginine (5 nM~5 μM) treatment for 72 h, suggesting that VA treatment could regulate arginine and its metabolites to promote MM cell proliferation.

### Elevated Ornithine Decarboxylase 1 Expression Is Associated With Poor Prognosis in MM

To gain further insights into the deregulated ornithine, we also explored the relationship between ODC1 known as the coding gene encoding ornithine decarboxylase (**Figure 6D**) and the prognosis of MM patients. GEP analysis showed that increased ODC1 expression was associated with poor overall survival (OS) in MM patients (TT2, GSE2658) (p=0.0002; **Figure 7E**). This result was also verified in the APEX phase III clinical trial with relapsed MM patients (p=0.0009; **Figure 7F**). Furthermore, analyses of two gene expression omnibus (GEO) databases, GSE5900 (p<0.0001; **Figure 7G**) and GSE6477 (p=0.0350; **Figure 7H**), demonstrated that ODC1 mRNA was significantly increased in MM patients compared with smoldering myeloma (SMM), monoclonal gammopathy of undetermined significance (MGUS), and normal plasma (NP).

## Discussion

Many clinical cases have shown the certain advantages of acupuncture and medicine combination in the treatment of pain (18, 19, 21, 22). Acupuncture can increase the number of white blood cells and alleviate leukopenia induced by radiotherapy (35). It was reported that acupuncture could reduce lymphedema and improve dyspnea symptoms in breast cancer patients (36, 37). In addition, acupuncture inhibits inflammation (38) and relieves symptoms caused by cancer *via* modulating vasomotion and stimulating the vagus nerve to modulate visceral inflammatory responses (39). Some clinical reports demonstrate that the acupuncture can be applied for symptom reduction in myeloma patients, including chemotherapy-induced peripheral neuropathy (40–42). However, there are few studies using metabolomics technology to find the therapeutic targets of MM. Our study first explored the mechanism of VA treatment for MM from the synergistic effect of VA treatment on MM and verified by *in vivo* experiment with metabolomics technology (**Figures 1A–C**).

We obtained the characteristics of metabolites in serum of MM model mice with either bortezomib or acupuncture or both treatments (**Figures 2A–H**, **3A–D**, **4A–L**). There were only 20 significantly upregulated metabolites (**Figure 5A**) and 32 significantly downregulated metabolites (**Figures 5B, E**) in group VA compared with control. Among them, ornithine as a unique downregulated metabolite in group VA was involved in the arginine and proline metabolic pathway, which showed the highest influence value (**Figures 5C, D**). Moreover, ornithine also participated in the regulation of glutathione metabolic pathway (43, 44), which may play a role during VA treatment. Therefore, it was suggested that ornithine might be a promising biomarker of VA therapy for MM (**Figures 6A–C**).

Arginine serving as a semi-essential amino acid possesses a significant impact on carcinogenesis and tumor biology (45), and it is mostly metabolized to ornithine by arginase (46, 47). Arginine metabolism is considered to be an important regulator in controlling immune response (48, 49), inhibiting antitumor immune response (50, 51), and promoting tumor development (34, 52). Ornithine is decarboxylated by ODC1 to produce putrescine, which is the rate-limiting step in polyamine biosynthesis (53, 54). Combined with cellular proliferation results (**Figures 7A–D**), we speculate that inhibiting arginine-ornithine metabolism can reduce ornithine content, thus decrease polyamine biosynthesis.

Last but not least, our data revealed that high ODC1 expression was significantly associated with poor prognosis in MM patients (**Figures 7E–H**). In fact, ODC1 is the exclusive gene encoding the rate-limiting enzyme of the polyamine biosynthesis pathway, which catalyzes ornithine to polyamines. Mounting studies reported that ODC1 expression was increased in many cancers, such as esophageal carcinoma (55), colorectal cancer (56), hepatocellular carcinoma (57), neuroblastoma (58), and ovarian cancer (59). Bianchi-Smiraglia A et al. (60) demonstrated that aryl hydrocarbon receptor (AHR) positively regulated intracellular polyamine production *via* direct transcriptional activation of ODC1 and AZIN1 genes, which inhibited the aryl hydrocarbon receptor/polyamine biosynthesis axis to suppress MM progression. Taken together, it may be concluded that combination of acupuncture and bortezomib can decrease ornithine and reduce ODC1 to prolong the survival time of MM. However, more work is needed to further validate the therapeutic effect of targeting arginine-ornithine metabolism and interfering ODC1 expression by using RNAi or difluoromethylornithine, an irreversible inhibitor of ornithine decarboxylase (61), to improve the effect of MM treatment.

In summary, our study demonstrates that combination of acupuncture and bortezomib has synergistic effects in the treatment of MM, which prolongs survival time of MM mice *via* decreasing ornithine. Targeting ornithine-mediated metabolism may be a promising way to benefit MM patients.

## Data Availability Statement

The original contributions presented in the study are included in the article/supplementary material. Further inquiries can be directed to the corresponding authors. The data presented in the study are deposited in the Metabolights repository, accession number MTBLS3487.

## Ethics Statement

The animal study was reviewed and approved by the Institutional Ethics Review Boards of Nanjing University of Chinese Medicine.

## Author Contributions

YY, CG, and BX designed the project, analyzed the data, and edited the manuscript. MK and JQ drafted the manuscript. MK, JQ, FH, XYL, HW, and XL performed the experimental work and analyzed the data. All authors contributed to the article and approved the submitted version.

## Funding

This work was supported by National Natural Science Foundation of China 81970196 (to CG) and 82073885 (to YY); Natural Science Foundation of Jiangsu Province BK20200097 (to CG); A Project Funded by the Priority Academic Program Development of Jiangsu Higher Education Institutions (Integration of Chinese and Western Medicine).

## Conflict of Interest

The authors declare that the research was conducted in the absence of any commercial or financial relationships that could be construed as a potential conflict of interest.

## Publisher’s Note

All claims expressed in this article are solely those of the authors and do not necessarily represent those of their affiliated organizations, or those of the publisher, the editors and the reviewers. Any product that may be evaluated in this article, or claim that may be made by its manufacturer, is not guaranteed or endorsed by the publisher.

## Abbreviations

3D, Three-dimensional; A, Acupuncture; APEX, the Assessment of Proteasome Inhibition for Extending Remissions; C, Control; CCK-8, Cell counting kit-8; ESI, Electron spray ionization; FC, Fold Change; GEO, Gene expression omnibus; GEP, Gene expression profiling; IMWG, International myeloma working group; IS, Internal standards; KEGG, Kyoto Encyclopedia of Genes and Genomes; LC-MS, Chromatography–mass spectrometry; MGUS, Monoclonal gammopathy of undetermined significance; MS, Mass spectrometric; NCCN, National comprehensive cancer network; NP, Normal plasma; ODC1, Ornithine decarboxylase 1; OPLS-DA, Partial Least Squares Discrimination Analysis; OS, Overall survival; PCA, Principal component analysis; rpm, Rotations per minute; RT, Retention time; SMM, Smoldering myeloma; TIC, Total ion current; TT2, Total therapy 2; V, Bortezomib; VA, Acupuncture combined with bortezomib; VIP, Variable importance in the projection.
